# Comparison of Surgical Outcomes in Robot-Assisted Nipple Sparing Mastectomy with Conventional Open Nipple Sparing Mastectomy: A Single Center Experience †

**DOI:** 10.3390/jcm14134608

**Published:** 2025-06-29

**Authors:** Ji Young You, Young Min Kim, Eun-shin Lee, Haemin Lee, Seung Pil Jung

**Affiliations:** 1 Division of Breast and Endocrine Surgery, Department of Surgery, Korea University Anam Hospital, Korea University College of Medicine, Seoul 02841, Republic of Korea; joliejean@korea.ac.kr (J.Y.Y.);; 2 Department of General Surgery, Inseong Medical Foundation Hallym Hospital, Incheon 14441, Republic of Korea

**Keywords:** breast neoplasm, nipple sparing mastectomy, robot-assisted surgery, da Vinci SP system, surgical outcomes, breast reconstruction, cancer resection

## Abstract

**Background**: A surgical therapy for breast cancer, robot-assisted nipple-sparing mastectomy (RANSM) has gained popularity because it may offer better cosmetic results than traditional nipple-sparing mastectomy (CNSM). Data regarding RANSM’s viability and security are still scarce, nevertheless. Comparing the surgical results of RANSM and CNSM in a single-center experience was the goal of this study. **Methods**: 57 patients who had nipple-sparing mastectomy procedures performed at our facility between January and December 2021 were included in this retrospective research. Of them, 49 patients had CNSM, and 8 patients had RANSM. Analysis was performed on pain scores, length of hospital stay, postoperative complications, patient demographics, and operating time. **Results**: The mean total operative time was longer for RANSM group was 148 min compared to 117 min for the CNSM group; however, this difference was not statistically significant (*p* = 0.083). The mean duration of hospital stay was shorter for the RANSM group than for the CNSM group (10.75 days vs. 2.92 days, respectively; *p* = 0.302). Both groups had similar pain scores on postoperative day 3 (RANSM: 3.50, CNSM: 3.54, *p* = 0.926). No patient in the RANSM group received adjuvant chemotherapy or radiotherapy, whereas 32.6% of patients in the CNSM group received chemotherapy. The RANSM and CNSM groups experienced complications at rates of 12.5% and 18.4%, respectively (*p* = 0.571). In contrast to 14.3% in the CNSM group, there were no documented incidences of skin necrosis in the RANSM group. **Conclusions**: RANSM demonstrated comparable safety to CNSM with potential benefits, including a shorter hospital stay and lower complication rates. These findings support the feasibility of RANSM, particularly in patients prioritizing cosmetic outcomes. To validate these initial findings, more research with larger cohorts and longer follow-up times is necessary.

## 1. Introduction

Early detection of breast cancer has increased recently, and neoadjuvant chemotherapy is given to patients with operable advanced breast cancer in order to increase the likelihood that surgery will be successful. According to this pattern, even in cases where entire resection is carried out, the goal of early breast cancer treatment has changed from merely minimizing the amount of surgery to enhancing patient satisfaction with better cosmetic results [1]. Nipple-sparing mastectomy (NSM), first reported by Freeman et al. in the 1960s [2,3], has been demonstrated to improve esthetic results and offer psychological advantages to patients with breast cancer, while achieving oncological safety comparable to that of a traditional mastectomy [4,5]. Although a variety of incision types, including radial, peri-areolar, elliptical, and inframammary incisions [6,7], are employed when performing NSM, the NSM approach is being extensively explored to achieve more discreet and effective incisions.

Endoscopic techniques were first used in the early 1990s to lessen the size of the incision and address the technical challenges of traditional incisional surgery as minimally invasive surgical techniques advanced [8,9]. However, the endoscopic approach was unpopular with surgeons owing to two-dimensional visualization and the lack of working space, unlike the abdominal cavity, which hindered instrument manipulation [10,11].

The most cutting-edge technology, the robotic surgical system, has been used extensively in the field of surgery for minimally invasive procedures. In comparison to traditional or endoscopic NSM, a number of studies have demonstrated that robot-assisted NSM (RANSM) is a technically viable and secure method [10,11,12]. Among them, the da Vinci SP^®^ system (Intuitive Surgical, Sunnyvale, CA, USA) is the latest robotic surgical system model that is more suitable for narrow spaces, enabling RNSM with minimal incision and without instrument collision. The surgeon can securely make a flap outside the breast dome with the aid of the cobra-like moving camera [13,14]. However, data on the safety and feasibility of robotic mastectomy remain limited.

The purpose of this study was to compare the surgical results and feasibility of RANSM with conventional NSM (CNSM) in patients with breast cancer.

## 2. Materials and Methods

### 2.1. Patient Selection

This retrospective observational study was conducted at a tertiary care center. Patients diagnosed with breast malignancy who underwent RANSM or CNSM, all with immediate breast reconstruction, at our institution between January 2021 and December 2021 were consecutively included in the study.

**Figure 1:**
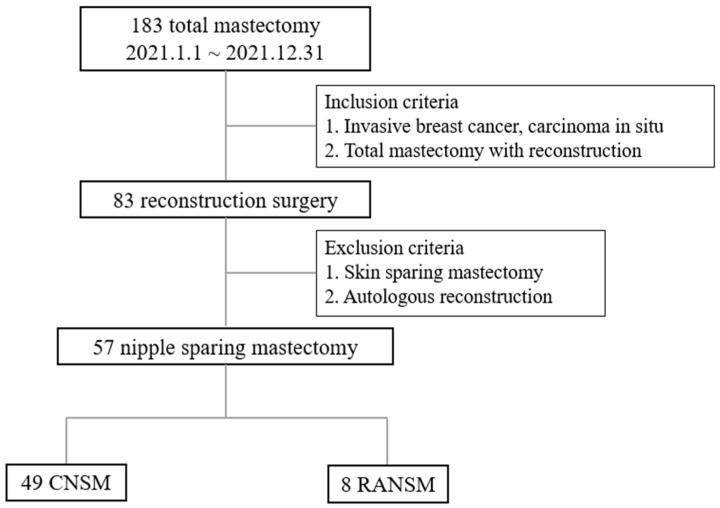
Study Flow.

Of the 183 patients who underwent total mastectomy for breast cancer, 83 underwent reconstruction surgery. The decision for mastectomy was based on multiple factors, such as tumor multifocality, presence of genetic mutations, patient preference, and prior history of radiation therapy. Due to possible variations in complication profiles, patients who had autologous reconstruction and those who had skin-sparing mastectomy, which is considered a relative contraindication for robotic surgery, were not included in the study [15]. In total, fifty-seven patients who underwent NSM were included in the final cohort: eight patients who underwent RANSM and forty-nine who underwent CNSM. The study flow is illustrated in Figure 1.

This retrospective study was authorized by the Institutional Review Board of our institution. The need for informed consent was waived.

### 2.2. Surgical Procedures of Robot-Assisted Nipple Sparing Mastectomy (RANSM)

**Figure 2:**
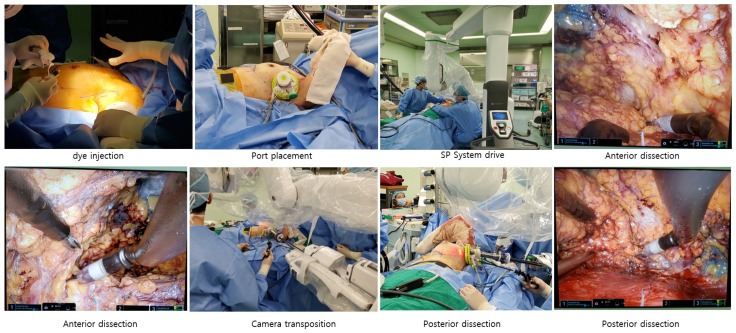
Surgical Procedures.

All robotic-assisted surgeries were performed using the da Vinci SP^®^ system. For RANSM, patients were positioned supine with the ipsilateral arm placed on an armboard. An incision was made at the nipple level that ran from the anterior to the mid-axillary line, measuring 4 to 5 cm. Prior to mastectomy, sentinel lymph node biopsy was performed manually using standard techniques via this incision. When ALND was necessary, it could be performed through the same robotic incision without difficulty. When additional exposure was required, the incision was extended by approximately 1 cm to allow for adequate access. Subcutaneous flap dissection was then carried out around the incision to create the necessary working space, followed by injection of blue dye and tumescent solution via a spinal tap needle. Subsequently, tunneling was performed using Metzenbaum scissors (Ethicon, Johnson & Johnson, Cincinnati, OH, USA), followed by the placement of the port and wound protector. The robotic system was subsequently docked, and CO_2_ insufflation was initiated. The remaining dissection was performed in an anterior-to-posterior direction using a monopolar curved scissor and bipolar ProGrasp forceps (Intuitive Surgical, Sunnyvale, CA, USA). To ensure oncologic safety, intraoperative frozen section of the nipple base was routinely performed, and resection margins were thoroughly evaluated. Once the breast parenchyma was completely detached, the specimen was retrieved through the incision (Figure 2). Immediate breast reconstruction was performed in all patients using a prepectoral implant placement technique. The reconstruction was conducted concurrently by the plastic surgery team during the same operative session. Implant type (anatomical or round silicone gel-filled) and size (ranging from 200 to 400 mL) were determined based on the patient’s breast dimensions and intraoperative evaluation.

### 2.3. Data Collection and Statistical Analysis

Clinicopathologic characteristics, including sex, age, body mass index (BMI), specimen weight, and preoperative diagnosis, were collected and analyzed. The following surgical outcomes were evaluated: the type of surgery (conventional or robotic), the duration of the operation (including docking and console time in robotic instances), the length of hospital stay, the postoperative complications, the pain scores, and if adjuvant local or systemic therapies were received. Data were categorized into two groups based on the surgical method: RANSM and CNSM. Retrospective analysis was performed on data related to demographics and surgical outcomes. Version 25 of IBM SPSS Statistics (IBM Corp., Armonk, NY, USA) was used to analyze the data. Means and standard deviations are used to convey continuous variables, whereas numbers and percentages are used to represent categorical variables. The chi-square (χ^2^) test or Fisher’s exact test, if applicable, was used to compare categorical data, whereas the independent t-test was used to analyze continuous variables. Statistical significance was defined as *p* < 0.05.

## 3. Results

Table 1 presents the demographic and clinical characteristics of patients who underwent the two types of surgeries. Forty-nine cases underwent CNSM (unilateral, *n* = 37 and bilateral, *n* = 12; the CNSM group), while eight cases underwent RANSM (unilateral, *n* = 6 and bilateral, *n* = 2; RANSM group). Both groups comprised female patients only. The mean patient age in the CNSM group was 48.3 years (range: 27–66 years) and was slightly higher in the RANSM group at 51.3 years (range: 38–60 years), although no statistically significant difference was noted (*p* = 0.843). The average specimen weight was 250.7 g (range: 110–580 g) in the CNSM and was slightly lower in the RANSM group at 215.6 g (range: 120–305 g), with no significant differences noted (*p* = 0.347). The BMI was comparable between the two groups: average BMI values for patients in the CNSM and RANSM groups were 21.46 kg/m^2^ (range: 14.9–35.82) and 22.33 kg/m^2^ (range: 18.89–26.28), respectively, with no significant difference detected (*p* = 0.495). Considering the diagnosis, the CNSM group comprised 33 cases of invasive ductal carcinoma, 3 cases of invasive lobular carcinoma, 9 cases of ductal carcinoma in situ (DCIS), 1 case of lobular carcinoma in situ (LCIS), 2 cases of mucinous carcinoma, and 1 case of papillary carcinoma. The RANSM group comprised 5 cases of invasive ductal carcinoma, 1 case of invasive lobular carcinoma, and 2 cases of DCIS. There were no cases of LCIS, mucinous, or papillary carcinoma in the RANSM group. Analysis of diagnostic categories revealed no statistically significant differences between the two groups (*p* = 0.974).

Regarding tumor stage, most patients in both groups presented with early-stage disease. In the CNSM group, Stage 0 was observed in 13 patients, Stage I in 17 patients, Stage II in 14 patients, and Stage III in 5 patients. In the RANSM group, 2 patients were classified as Stage 0, 5 as Stage I, and 1 as Stage II; no Stage III cases were identified. Although the RANSM group demonstrated a higher proportion of early-stage tumors, the difference was not statistically significant (*p* = 0.244).

In terms of molecular subtype distribution, the HR+/HER2− group was the most common subtype in both groups (CNSM: 38 cases; RANSM: 5 cases). HR+/HER2+ tumors were observed in 7 patients in the CNSM group and 2 patients in the RANSM group. HR−/HER2− (triple-negative) tumors were identified in 2 patients in the CNSM group and 1 patient in the RANSM group. The HR−/HER2+ subtype was not observed in either group. No significant difference was found in the distribution of molecular subtypes between the two groups (*p* = 0.470).

**Figure 3:**
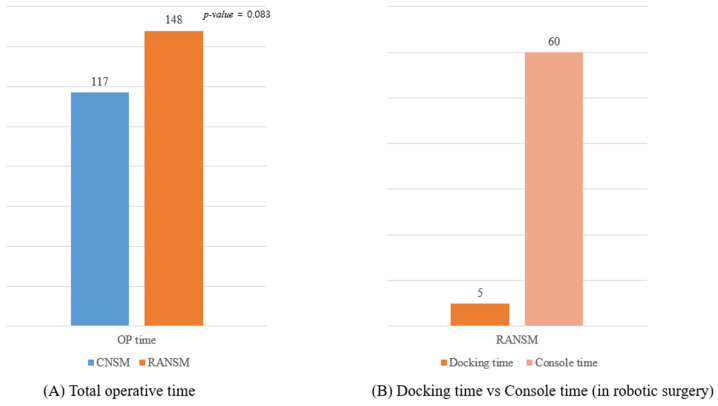
Operative Time.

Next, the mean total operative time, a crucial marker of procedural efficiency and surgical advancement, was compared between the CNSM and RANSM groups. The operative duration for RANSM was approximately 148 min, a substantial increase when compared with CNSM (117 min; Figure 3A). Despite the longer operative duration of RANSM, the *p*-value of 0.083 suggested that this difference was not statistically significant, indicating that while there is a trend toward longer operative time with robotic surgery, it may not be clinically relevant.

In the RANSM cohort, the breakdown of surgical times highlights a distinct difference between docking and console time (Figure 3B). The average docking time, a preparatory phase for the robotic system, is succinctly efficient, clocking in at a mere 5 min. In stark contrast, the console time, which encapsulates the period during which the surgeon manipulates the robotic controls to perform the surgical procedure, is markedly longer, averaging 60 min. Accordingly, the majority of time in RANSM is spent on the console during the procedure rather than on the setup. Overall, although RANSM requires more time than CNSM, this is largely due to the additional complexity and precision involved in the robotic approach.

Table 2 outlines the postoperative outcomes, demonstrating the absence of any statistically significant differences between the two groups. However, the RANSM group had a shorter mean hospital stay than the CNSM group (10.75 days vs. 12.92 days, respectively; *p* = 0.302). Postoperative pain, as measured using the numerical rating scale on postoperative day 3 (POD 3), was comparable between the two groups, with mean scores of 3.54 and 3.50 in the CNSM and RANSM groups, respectively (*p* = 0.926). Typically, the use of patient-controlled analgesics can act as a confounding factor until POD 2; hence, the pain score on POD 3 was used as a standardized index for precise comparison.

Notably, none of the patients in the RANSM group received adjuvant chemotherapy or radiotherapy, whereas 32.6% of patients in the CNSM group underwent chemotherapy, with 20.4% receiving it as an adjuvant therapy. The incidence of postoperative complications was similar between the two groups, occurring in 18.4 and 12.5% of patients in the CNSM and RANSM groups, respectively (*p* = 0.571). Skin necrosis was observed in 14.3% of patients in the CNSM group, while no cases of skin necrosis were documented in the RANSM group. However, one patient experienced capsular contracture in the RANSM group, a complication not observed in the CNSM group.

## 4. Discussion

In this study, we compared the surgical outcomes of RANSM with those of CNSM in patients with breast cancer. Our results demonstrated that RANSM could be a feasible and safe procedure with potential advantages over CNSM despite some limitations.

The mean operative time for RANSM was longer than that for CNSM (148 min vs. 117 min); however, this difference was not statistically significant (*p* = 0.083). This increased duration is primarily attributed to the console time (average 60 min) rather than the docking time (average 5 min). Our findings align with those of a recent meta-analysis that reported a notable increase in operating time for robotic surgeries [12,16]. The extended operative time in RANSM likely reflects the learning curve associated with new technology [17]. Loh et al. analyzed the learning curve for RANSM using cumulative sum plots, demonstrating a substantial decrease in mastectomy time after the 22nd procedure [18]. This finding suggests that with increased experience, the operative time for RANSM is likely to decrease [14,19].

Although statistically non-significant, patients who underwent RANSM in our study had a shorter mean hospital stay than those who underwent CNSM (10.75 days vs. 12.92 days, respectively; *p* = 0.302). This trend contradicts findings from the meta-analysis by Nessa et al., which reported a significantly longer length of hospital stay for robotic procedures (mean difference +1.23 days, *p* < 0.0001) [16]. This discrepancy warrants further investigation and may be attributed to differences in postoperative care protocols or patient selection criteria.

The complication rates were similar between the two groups in our study (12.5% for RANSM vs. 18.4% for CNSM, *p* = 0.571). Notably, no skin necrosis was observed in the RANSM group, compared to 14.3% in the CNSM group. This aligns with the meta-analysis findings, which reported a significant reduction in nipple necrosis with robotic procedures [16,20].

The lower incidence of skin and nipple necrosis in the RANSM group may be attributed to enhanced visualization and precise dissection afforded by the robotic system.

Our study did not observe any local recurrences during the follow-up period. However, definitive conclusions regarding oncological safety cannot be drawn owing to the short-term nature of our follow-up. Moreover, Nessa et al.’s systematic study highlights the necessity of a thorough evaluation of robotic breast surgery’s long-term oncological results [16].

Finally, it is important to recognize a number of this study’s shortcomings. First, the comparison’s statistical power was constrained by the small sample size, especially in the RANSM group (*n* = 8). According to Loh et al. and Toesca et al. [18,21], this is a typical limitation in early-phase research assessing novel surgical procedures, and the learning curve evaluation for RANSM in this work revealed comparable results. Second, there may be selection bias, particularly among patients who choose nipple-sparing mastectomy since they frequently value cosmetic results and, hence, have favorable tumor biology and very early-stage cancer. Consequently, the percentage of patients undergoing adjuvant chemotherapy was lower than that of patients with breast cancer in general. Third, just two surgeons carried out the RANSM procedures in this study, which might not accurately represent the wider learning curve involved in implementing novel robotic surgical techniques. As additional examples are gathered, we intend to perform surgeon-specific analysis because the learning curve may differ greatly when various surgeons are involved. Results from future multicenter studies with additional surgeons would be more broadly applicable. Lastly, in order to properly evaluate the clinical efficacy of RANSM, this study did not evaluate cost-effectiveness or long-term oncologic results. The need for more research in these areas is shown by the contradictory results of various earlier papers that looked at cost analyses and cosmetics [22,23].

## 5. Conclusions

Although RANSM requires a longer operative time than CNSM, its potential advantages in terms of reduced complications, particularly nipple necrosis, suggest that it could become an increasingly preferred method for NSMs. As we continue to refine this technique and accumulate additional data, RANSM may play a substantial role in the future of breast cancer surgery, offering patients the benefits of minimally invasive surgery without compromising oncologic safety. To further understand the role of robotic surgery in the treatment of breast cancer, future research should examine patient-reported outcomes, cost-effectiveness, and long-term oncologic safety using larger cohorts.

## Figures and Tables

**Table 1:** Patent Demographics.

		CNSM	RANSM	*p*-Value
Case		49 (unilateral 37, bilateral 12)	8 (unilateral 6, bilateral 2)	N/A
Sex		All Female	All Female	N/A
Age		48.3 (27~66)	51.3 (38~60)	0.843
Breast Weight (g)		250.7 (110~580)	215.6 (120~305)	0.347
BMI (kg/m^2^)		21.46 (14.9~35.82)	22.33 (18.89~26.28)	0.495
Diagnosis	IDC	33	5	0.974
	ILC	3	1	
	DCIS	9	2	
	LCIS	1	0	
	Mucinous	2	0	
	Papillary	1	0	
Stage	Stage 0	13	2	0.244
	Stage I	17	5	
	Stage II	14	1	
	Stage III	5	0	
Subtype *	HR (+) HER2 (−)	38 (8)	5 (1)	0.470
	HR (+) HER2 (+)	7 (2)	2 (0)	
	HR (−) HER2 (+)	2 (2)	0	
	HR (−) HER2 (−)	2 (1)	1 (1)	

* Values in parentheses represent Stage 0 cases.

**Table 2:** Postoperative Outcomes.

	CNSM	RANSM	*p*-Value
Mean Hospital stay (day)	12.92	10.75	0.302
POD 3 pain score (NRS *)	3.54	3.50	0.926
Chemotherapy (%)	16 (32.6%)	0 (0%)	0.058
Neoadjuvant	6 (12.2%)	0 (0%)	
Adjuvant	10 (20.4%)	0 (0%)	
Radiotherapy	4 (8.2%)	0 (0%)	0.536
Complication	9 (18.4%)	1 (12.5%)	0.571
Skin necrosis	7 (14.3%)	0 (0%)	
Capsule contraction	0 (0%)	1 (12.5%)	
Infection	2 (4.1%)	0 (0%)	

* NRS: Numeric rating scroe (0–10).

## Data Availability

The data that support the findings of this study are available from the corresponding author upon reasonable request. The data are not publicly available due to privacy and ethical restrictions, as they contain information that could compromise the privacy of research participants.

## Abbreviations

The following abbreviations are used in this manuscript:

CNSM	conventional nipple sparing mastectomy
RANSM	robot-assisted nipple sparing mastectomy
IDC	invasive ductal carcinoma
ILC	invasive lobular carcinoma
DCIS	ductal carcinoma in situ
LCIS	lobular carcinoma in situ

## References

[1] Murugappan K., Saboo A., Kuo L., Ung O. (2018). Paradigm shift in the local treatment of breast cancer: Mastectomy to breast conservation surgery. Gland Surg..

[2] Freeman B.S. (1962). Subcutaneous mastectomy for benign breast lesions with immediate or delayed prosthetic replacement. Plast. Reconstr. Surg. Transplant Bull..

[3] Freeman B.S. (1967). Complications of subcutaneous mastectomy with prosthetic replacement, immediate or delayed. South Med. J..

[4] Gerber B., Krause A., Dieterich M., Kundt G., Reimer T. (2009). The oncological safety of skin sparing mastectomy with conservation of the nipple-areola complex and autologous reconstruction: An extended follow-up study. Ann. Surg..

[5] Orzalesi L., Casella D., Santi C., Cecconi L., Murgo R., Rinaldi S., Regolo L., Amanti C., Roncella M., Serra M. (2016). Nipple sparing mastectomy: Surgical and oncological outcomes from a national multicentric registry with 913 patients (1006 cases) over a six year period. Breast J..

[6] Blechman K.M., Karp N.S., Levovitz C., Guth A.A., Axelrod D.M., Shapiro R.L., Choi M. (2013). The lateral inframammary fold incision for nipple-sparing mastectomy: Outcomes from over 50 immediate implant-based breast reconstructions. Breast J..

[7] Cavalcante F.P., Lima M.V.A. (2018). Nipple-sparing mastectomy with periareolar incision and two-stage reconstruction: Initial analysis of 31 cases. Breast J..

[8] Nakajima H., Fujiwara I., Mizuta N., Sakaguchi K., Hachimine Y. (2009). Video-assisted skin-sparing breast-conserving surgery for breast cancer and immediate reconstruction with autologous tissue. Ann. Surg..

[9] Park H.S., Lee J.S., Lee J.S., Park S., Kim S.I., Park B.W. (2011). The feasibility of endoscopy-assisted breast conservation surgery for patients with early breast cancer. J. Breast Cancer.

[10] Leff D.R., Vashisht R., Yongue G., Keshtgar M., Yang G.Z., Darzi A. (2011). Endoscopic breast surgery: Where are we now and what might the future hold for video-assisted breast surgery?. Breast Cancer Res. Treat..

[11] Lai H.W., Chen S.T., Tai C.M., Lin S.L., Lin Y.J., Huang R.H., Mok C.W., Chen D.R., Kuo S.J. (2020). Robotic- Versus Endoscopic-Assisted Nipple-Sparing Mastectomy with Immediate Prosthesis Breast Reconstruction in the Management of Breast Cancer: A Case-Control Comparison Study with Analysis of Clinical Outcomes, Learning Curve, Patient-Reported Aesthetic Results, and Medical Cost. Ann. Surg. Oncol..

[12] Angarita F.A., Castelo M., Englesakis M., McCready D.R., Cil T.D. (2020). Robot-assisted nipple-sparing mastectomy: Systematic review. Br. J. Surg..

[13] Park H.S., Lee J., Lee H., Lee K., Song S.Y., Toesca A. (2020). Development of Robotic Mastectomy Using a Single-Port Surgical Robot System. J. Breast Cancer.

[14] Houvenaeghel G., Bannier M., Rua S., Barrou J., Heinemann M., Van Troy A., Lambaudie E., Cohen M. (2019). Breast cancer robotic nipple sparing mastectomy: Evaluation of several surgical procedures and learning curve. World J. Surg. Oncol..

[15] Lai H.W., Toesca A., Sarfati B., Park H.S., Houvenaeghel G., Selber J.C., Cheng F.T.F., Kuo W.L., Peradze N., Song S.Y. (2020). Consensus Statement on Robotic Mastectomy-Expert Panel From International Endoscopic and Robotic Breast Surgery Symposium (IERBS) 2019. Ann. Surg..

[16] Nessa A., Shaikh S., Fuller M., Masannat Y.A., Kastora S.L. (2024). Postoperative complications and surgical outcomes of robotic versus conventional nipple-sparing mastectomy in breast cancer: Meta-analysis. Br. J. Surg..

[17] Sarfati B., Honart J.F., Leymarie N., Rimareix F., Al Khashnam H., Kolb F. (2018). Robotic da Vinci Xi-assisted nipple-sparing mastectomy: First clinical report. Breast J..

[18] Loh Z.J., Wu T.Y., Cheng F.T. (2021). Evaluation of the Learning Curve in Robotic Nipple-sparing Mastectomy for Breast Cancer. Clin. Breast Cancer.

[19] Park H.S., Lee J., Lee D.W., Song S.Y., Lew D.H., Kim S.I., Cho Y.U. (2019). Robot-assisted Nipple-sparing Mastectomy with Immediate Breast Reconstruction: An Initial Experience. Sci. Rep..

[20] Park H.S., Lee J., Lai H.-W., Park J.M., Ryu J.M., Lee J.E., Kim J.Y., Marrazzo E., De Scalzi A.M., Corso G. (2022). Surgical and Oncologic Outcomes of Robotic and Conventional Nipple-Sparing Mastectomy with Immediate Reconstruction: International Multicenter Pooled Data Analysis. Ann. Surg. Oncol..

[21] Toesca A., Peradze N., Galimberti V., Manconi A., Intra M., Gentilini O., Sances D., Negri D., Veronesi G., Rietjens M. (2017). Robotic Nipple-sparing Mastectomy and Immediate Breast Reconstruction with Implant: First Report of Surgical Technique. Ann. Surg..

[22] Lai H.W., Chen D.R., Liu L.C., Chen S.T., Kuo Y.L., Lin S.L., Wu Y.C., Huang T.C., Hung C.S., Lin Y.J. (2024). Robotic Versus Conventional or Endoscopic-assisted Nipple-sparing Mastectomy and Immediate Prosthesis Breast Reconstruction in the Management of Breast Cancer: A Prospectively Designed Multicenter Trial Comparing Clinical Outcomes, Medical Cost, and Patient-reported Outcomes (RCENSM-P). Ann. Surg..

[23] Lai H.-W., Chen S.-T., Lin S.-L., Chen C.-J., Lin Y.-L., Pai S.-H., Chen D.-R., Kuo S.-J. (2019). Robotic Nipple-Sparing Mastectomy and Immediate Breast Reconstruction with Gel Implant: Technique, Preliminary Results and Patient-Reported Cosmetic Outcome. Ann. Surg. Oncol..

[24] You J.Y., Lee H.M., Lee E.S., Jung S.P. Comparison of robot-assisted nipple sparing mastectomy with conventional nipple sparing mastectomy: A single-center experience during a recent year. Proceedings of the San Antonio Breast Cancer Symposium.

